# Supplementary material for: A polytherapy based approach to combat antimicrobial resistance using cubosomes
Source: Nat Commun. 2022 Jan 17;13:343. doi: 10.1038/s41467-022-28012-5 (PMC8763928; doi:10.1038/s41467-022-28012-5)
Supplement: Supplementary file 1 — Supplementary Information [file 41467_2022_28012_MOESM1_ESM.pdf]

Supplementary Information

A polytherapy based approach to combat antimicrobial resistance using cubosomes

Xiangfeng Lai[1], Mei-Ling Han[2], Yue Ding[1,3], Seong Hoong Chow[3], Anton P. Le Brun[4], Chun-Ming Wu[4,5], Philip J. Bergen[2], Jhih-hang Jiang[2], Hsien-Yi Hsu[6,7], Benjamin W. Muir[8], Jacinta White[8], Jiangning Song[3], Jian Li [2]*and Hsin-Hui Shen[1,3]*

[1]Department of Materials Science and Engineering, Faculty of Engineering, Monash University, Clayton, Victoria 3800, Australia

[2]Infection and Immunity Program, Monash Biomedicine Discovery Institute and Department of Microbiology, Monash University, Clayton, VIC 3800, Australia

[3]Biomedicine Discovery Institute and Department of Biochemistry and Molecular Biology, Monash University, Clayton, Victoria 3800, Australia

[4]Australian Centre for Neutron Scattering, Australian Nuclear Science and Technology Organisation, Locked Bag 2001, Kirrawee DC, New South Wales 2232, Australia

[5]National Synchrotron Radiation Research Center, Hsinchu 30076, Taiwan

[6]School of Energy and Environment & Department of Materials Science and Engineering, City University of Hong Kong, Kowloon Tong, Hong Kong, China

[7]Shenzhen Research Institute of City University of Hong Kong, Shenzhen 518057, China

[8]CSIRO Manufacturing, Clayton, Victoria 3168 Australia

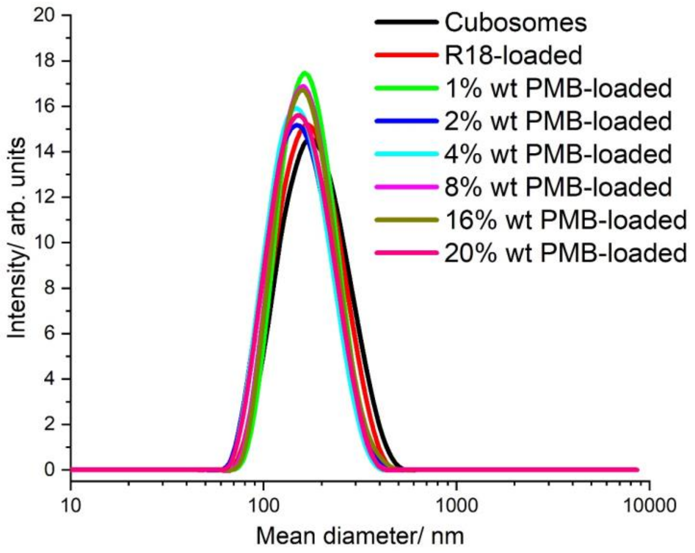

Supplementary Fig. 1: DLS profiles of cubosomes, R18-loaded and PMB-loaded cubosomes. Results are summarized in **Supplementary Table 1**. The intensity distribution is weighted according to the scattering intensity of each particle fraction. arb. units, arbitrary unit. Source data are provided as a Source Data file.

Supplementary Table 1: Particle properties of Pure phytantriol cubosomes, R18-loaded and 1-20wt% PMB-loaded cubosomes.

Samples	Particle size (d, nm)	Polydispersity index (PDI)	zeta potential (mV)
Pure phytantriol cubosomes	172.6 ± 7.2	0.10 ± 0.01	-23.8 ± 1.0[a]
R18-loaded cubosomes	162.7 ± 3.9	0.11 ± 0.02	-19.6 ± 0.8
1 wt % PMB-loaded cubosomes	165.8 ± 9.2	0.16 ± 0.05	-17.0 ± 1.0
2 wt % PMB-loaded cubosomes	149.0 ± 2.3	0.14 ± 0.01	-15.1 ± 0.9
4 wt % PMB-loaded cubosomes	149.0 ± 2.7	0.16 ± 0.04	-12.3 ± 1.0
8 wt % PMB-loaded cubosomes	154.5 ± 0.8	0.09 ± 0.02	-1.3 ± 0.4
16 wt % PMB-loaded cubosomes	159.7 ± 12.2	0.13 ± 0.02	1.2 ± 0.7
20 wt % PMB-loaded cubosomes	147.1 ± 1.7	0.11 ± 0.01	1.6 ± 0.2

[a]The negative zeta potential of cubosomes could be due to the incorporation of negatively charged F127[1]. Data are expressed as mean ± standard deviation (S.D.). All experiments were performed in triplicate (n=3).

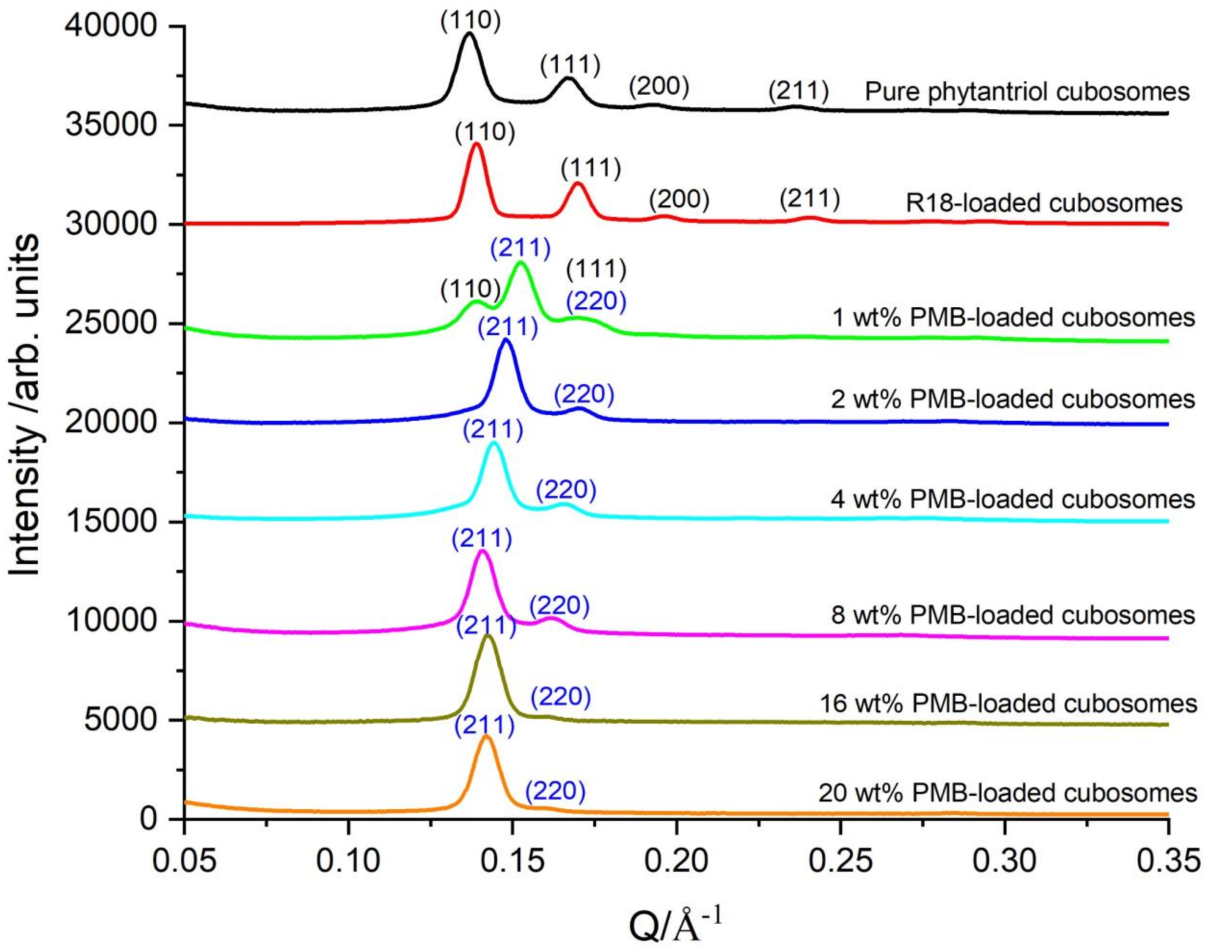

Supplementary Fig. 2. **SAXS profiles of Pure phyatnriol cubosomes, R18-loaded and 1-20 wt% PMB-loaded cubosome dispersions in MilliQ-water.** The structures of the nanoparticles were assigned by the calculation from the Bragg peaks, where the relative positions of the first two peaks in ratios of $\sqrt{2} : \sqrt{4}$ correspond to *Pn3m* symmetry, while $\sqrt{6} : \sqrt{8}$ correspond to Ia3d symmetry[2,3]. arb. units, arbitrary unit. Source data are provided as a Source Data file.

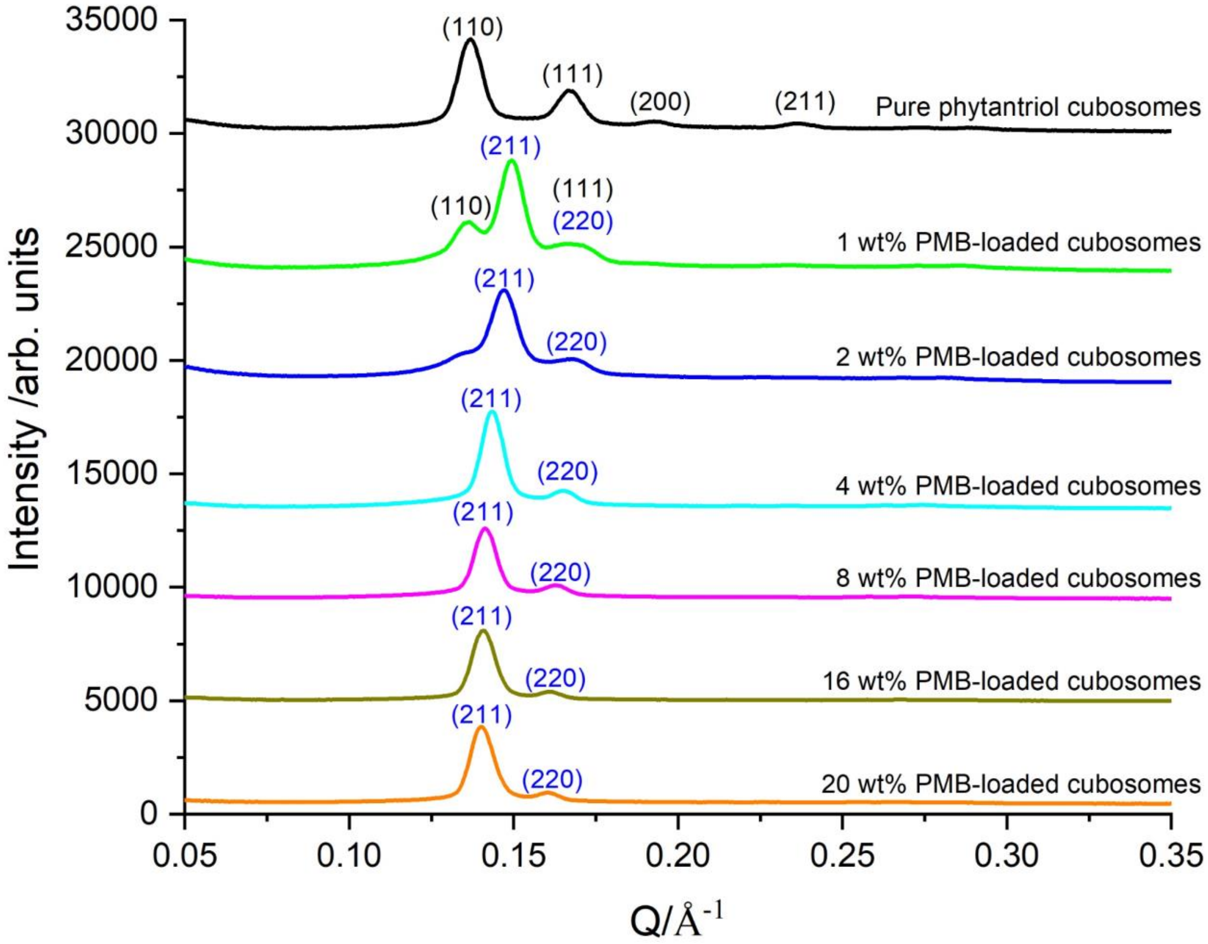

Supplementary Fig. 3. **SAXS profiles of pure phatantriol cubosomes and 1-20 wt% PMB-loaded cubosome dispersions in PBS buffer**. The consistency of the SAXS curves in between MilliQ water and PBS buffer suggests the structural stability of the nanoparticles upon probe sonication in these two solutions. arb. units, arbitrary unit. Source data are provided as a Source Data file.

Supplementary Table 2. Phase structures, unit cell parameters and water channel radius of pure phytantriol cubosomes, R18-loaded and 1 – 20 wt% PMB-loaded cubosomes derived from **Supplementary Figure 2-3**.

Nanoparticles	Space group	unit cell parameter (a, nm)	$r_w{}^a$ (nm)
Cubosomes	$Q_{II}{}^D$ (*Pn3m*)	6.51 ± 0.02	1.15 ± 0.01
R18-loaded	$Q_{II}{}^D$ (*Pn3m*)	6.39 ± 0.01	1.10 ± 0.01
1 wt% PMB-loaded	$Q_{II}{}^D$ (*Pn3m*) + $Q_{II}{}^G$ (*Ia3d*)	N/A	N/A
2 wt% PMB-loaded	$Q_{II}{}^G$ (*Ia3d*)	10.69 ± 0.06	1.25 ± 0.02
4 wt% PMB-loaded	$Q_{II}{}^G$ (*Ia3d*)	10.85 ± 0.03	1.29 ± 0.01
8 wt% PMB-loaded	$Q_{II}{}^G$ (*Ia3d*)	11.12 ± 0.07	1.36 ± 0.02
16 wt% PMB-loaded	$Q_{II}{}^G$ (*Ia3d*)	10.75 ± 0.02	1.27 ± 0.01
20 wt% PMB-loaded	$Q_{II}{}^G$ (*Ia3d*)	11.01 ± 0.06	1.33 ± 0.02

[a]Radius of water channel; The Bonnet ratios of unit cell parameters of *Ia3d*: *Pn3m*: *Im3m* is 1.58: 1.00: 1.28[4]. The unit cell parameter ratio of 2-20 wt % PMB-loaded cubosomes to cubosomes is 1.64, which agrees with the Bonnet ratio values (1.58), so we can exclude *Im3m* symmetry. N/A, not available.

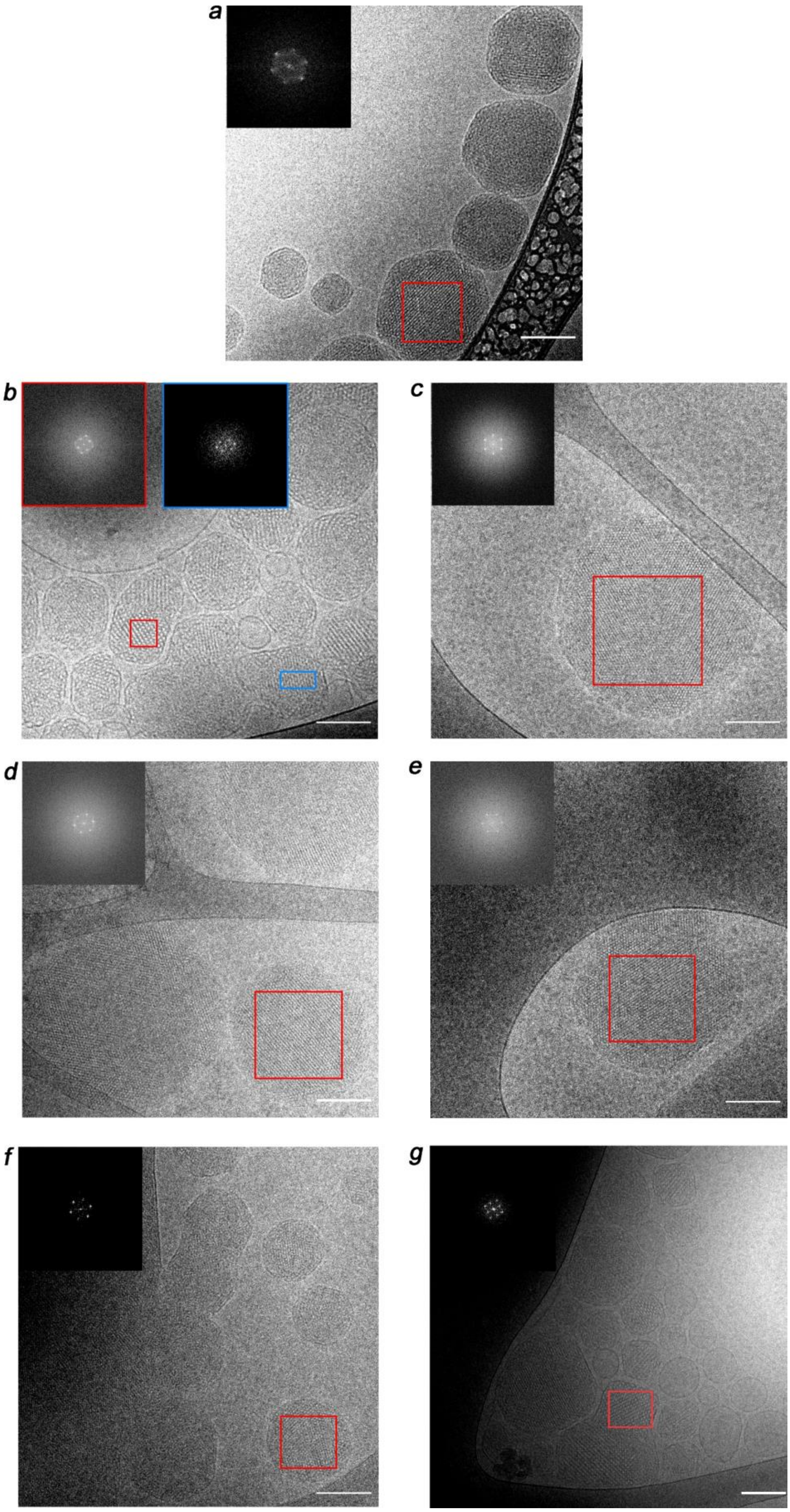
a
b
c
d
e
f
g

Supplementary Fig. 4. **Cryo-TEM images of 0-20 (w/w) % PMB-loaded cubosomes, the inserts display Fast Fourier Transform (FFT) analysis of the corresponding dispersed particles. a**, phytantriol cubosomes; **b**, 1 wt % PMB-loaded cubosomes; **c**, 2 wt % PMB-loaded cubosomes; **d**, 4 wt % PMB-loaded cubosomes; **e**, 8 wt % PMB-loaded cubosomes; **f**, 16 wt % PMB-loaded cubosomes; and **g**, 20 wt % PMB-loaded cubosomes. All the images are representative of experiments performed in triplicate (n = 3). Scale bar: 100 nm.

Supplementary Table 3. PMB entrapment efficiency of PMB-loaded cubosomes.

PMB-loaded cubosomes	Entrapment efficiency (%)
1 wt% PMB	98.6 ± 0.1
2 wt% PMB	97.3 ± 0.2
4 wt% PMB	96.0 ± 0.1
8 wt% PMB	94.8 ± 0.2
16 wt% PMB	95.9 ± 0.1
20 wt% PMB	94.4 ± 0.1

All data were expressed as mean ± S.D. All experiments were performed in triplicate (n=3). Source data are provided as a Source Data file.

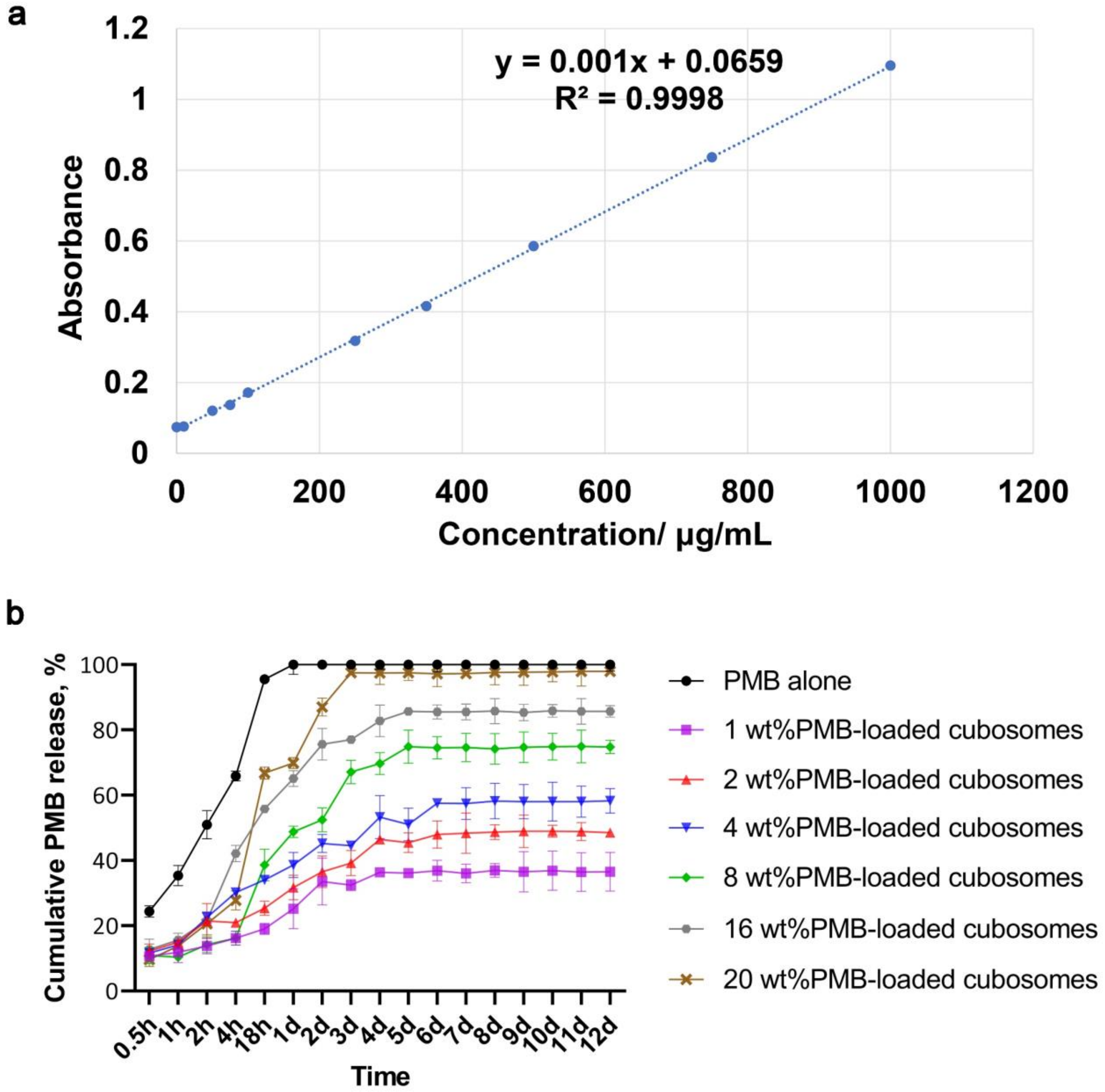

Supplementary Fig. 5. **PMB release profile from cubosomes. a**, Analytical curve of PMB by UV/Vis spectrophotometry, λ = 210 nm. **b**, PMB *in vitro* release from PMB-loaded cubosomes compared to PMB aqueous solution (PMB in dialysis bag as PMB-release control) in PBS solutions at 37 °C presented as the individual amount of released PMB *vs* time (up to 12 days). All Data in (**b**) are expressed as the mean ± S.D. All experiments were performed in biologically triplicate (n=3). Mean values and error bars were defined as mean and S.D., respectively. Source data are provided as a Source Data file.

Supplementary Table 4. Fractional inhibitory concentrations (FICs) of PMB with 8 wt% PMB-loaded cubosomes against isolates of *A. baumannii*, *P. aeruginosa*, and *K. pneumoniae* in CaMHB.

Bacteria	Treatment	FIC[a] (μg/mL)
A. baumannii ATCC 19606	PMB/8 wt% PMB	0.17±0.06/3.33±0.94
A. baumannii 5075	PMB/8 wt% PMB	2.00[b]/0.58±0.31
A. baumannii FADDI-AB240	PMB/8 wt% PMB	0.25 [b]/13.3±3.77
A. baumannii FADDI-AB241	PMB/8 wt% PMB	8.00[b]/0.21±0.06
P. aeruginosa FADDI-PA070	PMB/8 wt% PMB	16.00[b]/0.58±0.31
K. pneumoniae ATCC 700721	PMB/8 wt% PMB	0.13[b]/16.00[b]
K. pneumoniae FADDI-KP012	PMB/8 wt% PMB	4.00[b]/0.33±0.12

[a]Experiments were undertaken immediately after the preparation of 8 wt% MB-loaded cubosomes; [b]Values were identical across all experiments; All data were expressed as mean ± S.D. All experiments were performed in triplicate (n=3). Names of all bacterial taxa are printed in italics.

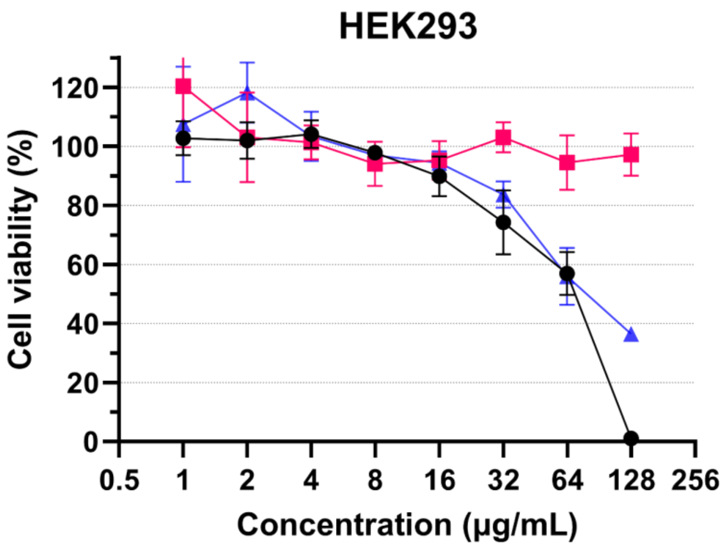

Supplementary Fig. 6. ***In vitro* cell viability of HEK293T cells incubated in the presence of cubosomes, PMB and PMB/cubosomes after 24 h of incubation.** Cell viability measurements are given relative to those of control samples (HEK293 cells incubated in the absence of treatment) (Error bars = S.D.). All Data are expressed as the mean ± S.D. All experiments were performed in triplicate (n=3). Mean values and error bars were defined as mean and S.D., respectively. Black circle, cubosome treatment; red square, PMB treatment; blue up triangle, polytherapy of PMB and cubosomes. Source data are provided as a Source Data file.

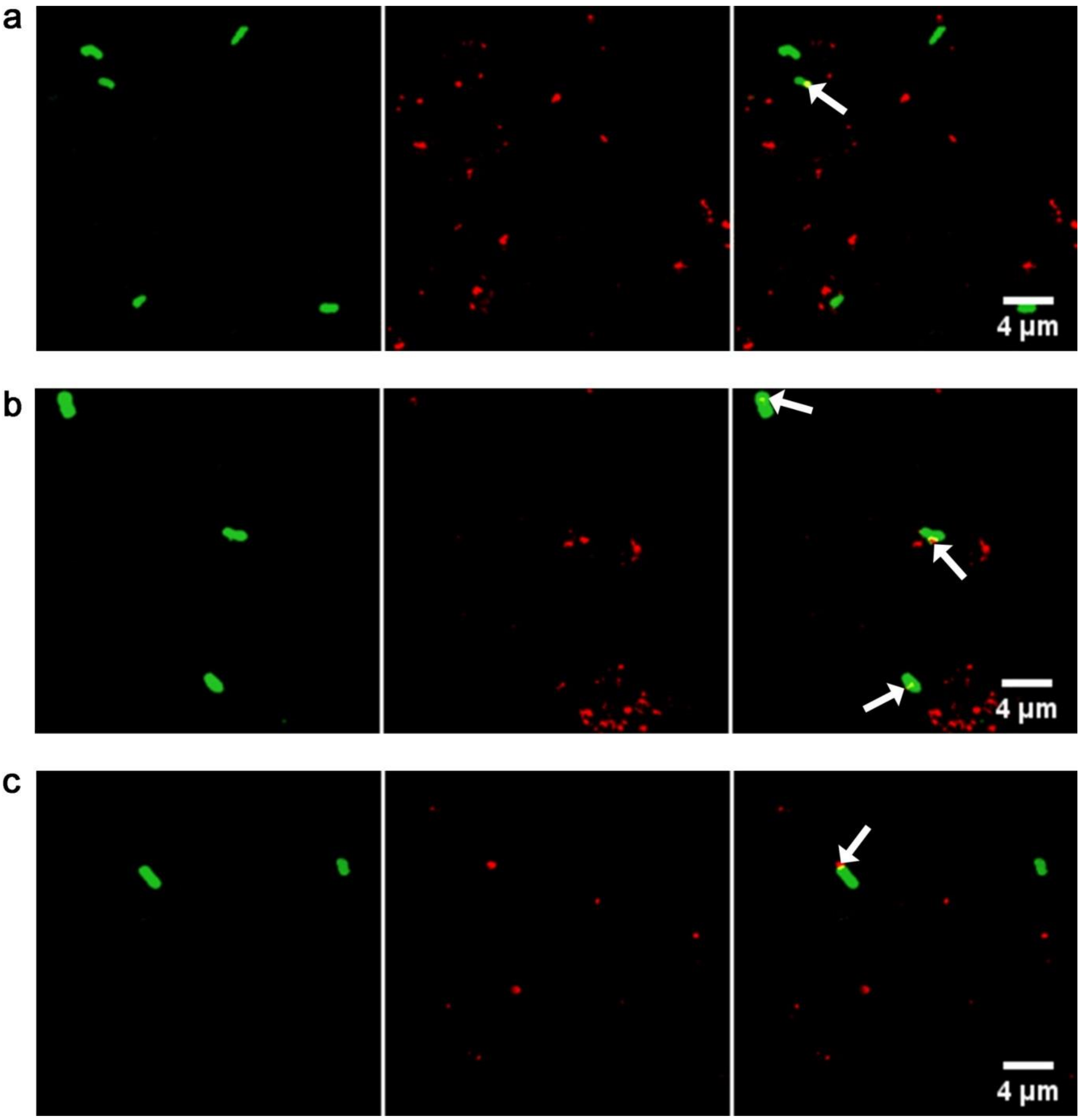

Supplementary Fig. 7. Representative fluorescent microscopy images of *Ab* ATCC 19606. Treatment with **a**, 2 wt % PMB-loaded cubosomes; **b**, 4 wt % PMB-loaded cubosomes; and **c**, 16 wt % PMB-loaded cubosomes. After PMB-loaded cubosome treatment, the overlays of the respective micrographs were observed (arrows), however the cubosomes were found to be merely associated on the surface of the bacteria, indicating the week interaction of cubosomes with the bacterial membrane. All the images are representative of experiments performed in triplicate (n = 3). Left: bacteria in green; Middle: cubosomes in red; Right: overlay image.

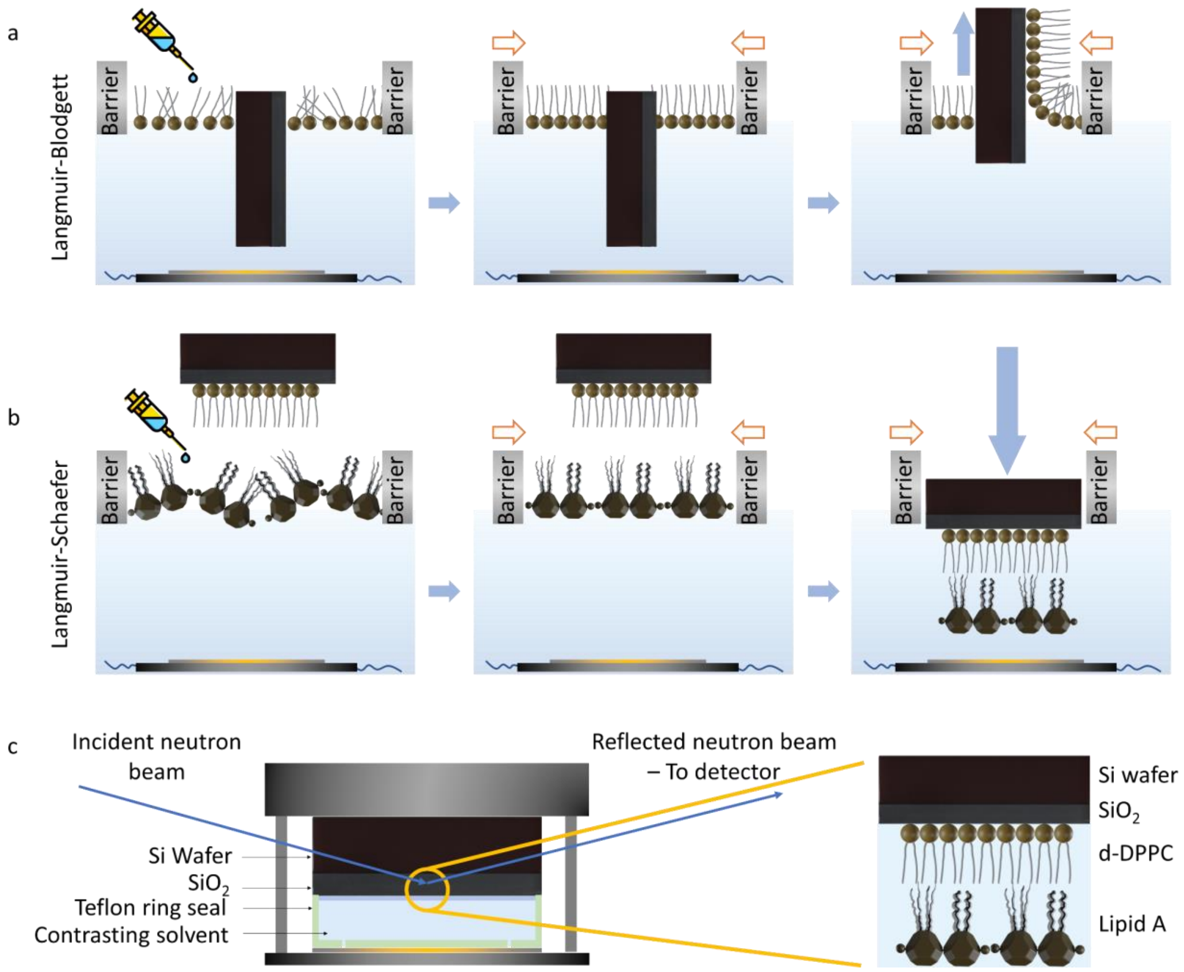

Supplementary Fig. 8. Schematic representation of the LB and LS depositions. Initially, silicon wafer was washed with 2% (v/v) Hellmanex solution and Milli-Q water repeatedly before incubation in an ozone chamber for 15 min to clean the silicon dioxide (SiO_2) surface. The ozone-cleaned wafer was then submerged into the LB trough filled with 5 mM $CaCl_2$ solution at 10°C. **a**, After the surface pressure reached 0 mN/m by vacuum-pump cleaning, d-DPPC dissolved in chloroform at a final concentration of 2 mg/mL was slowly added via a micro syringe and carefully spread onto the liquid surface. After complete evaporation of the chloroform, the pressure control program was initiated by means of two barriers located at the interface to compress the d-DPPC monolayer until the surface pressure sensor reached a pre-set packing density (constant surface pressure of 37 mN/m). Once the monolayer was stably fabricated in the air-liquid interface, the Langmuir film was deposited on the hydrophilic SiO_2 by raising the wafer from the sub-phase through the monolayer. **b**, The liquid surface was vacuum-cleaned to thoroughly remove the d-DPPC residues. The wafer was then rotated by 90° parallel to the water surface which was measured by the built-in laser levelling device. Lipid A dissolved in chloroform at a

concentration of 2 mg/mL was then added in the same way onto the surface and compressed to a constant surface pressure of 37 mN/m. The wafer was crept down through the air-liquid interface parallel to the d-DPPC monolayer to form the final asymmetrical bilayer and lowed into a cell of submerged silicon base. **c**, Finally, the aluminium thermostatic cage was assembled under the liquid surface and sent to the NR instrument for examination. Neutrons reflect at interfaces present inside the cell, such as the Si wafer/SiO_2 interface, SiO_2/lipid interface and the lipid/water interface. The reflectivity profile of the adsorbed membrane bilayer is generated by the interference between the neutrons reflected from the different interfaces.

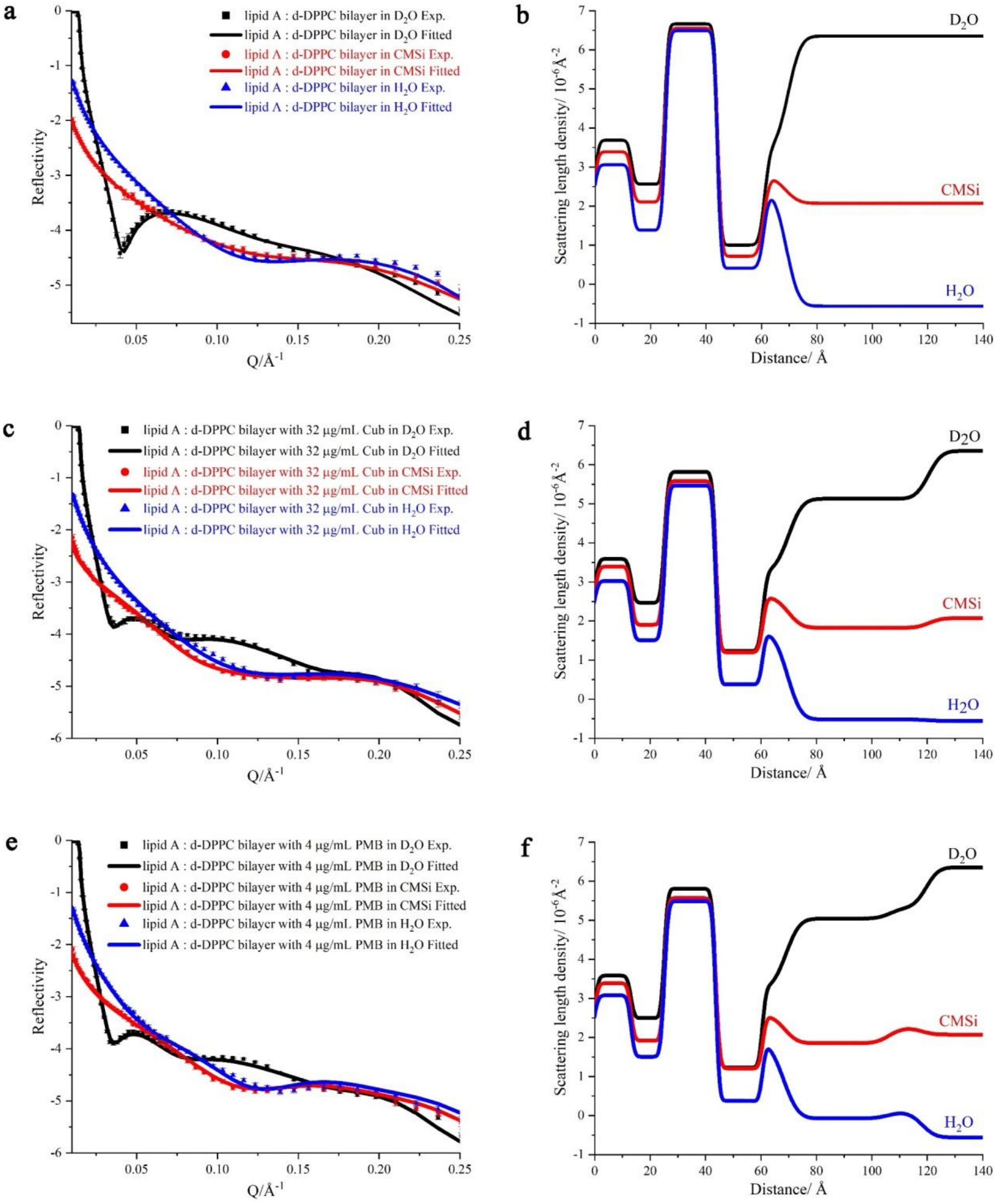

1. **Supplementary Fig. 9**. **Lipid A: d-DPPC bilayer treated with 32 µg/mL cubosomes followed by 4 µg/mL PMB.** Experimental (Exp., symbols) and fitted (solid lines) NR profiles in D_2O (black squares), CMSi (red circles) and H_2O (blue up triangle). **a**, lipid A: d-DPPC bilayer; **b**, the corresponding SLD profiles of (**a**); **c**, bilayer (**a**) treated with 32 µg/mL cubosomes (Cub); **d**, the corresponding SLD profiles of (**c**); **e**, followed by treatment with 4 µg/mL PMB; **f**, the corresponding SLD profiles of (**e**). For each condition in neutron reflectometry, we conducted multiple isotopic contrasts (D_2O, H_2O and CMSi) which would remove any artifacts in the fitting. The error bars are the standard deviation of neutron counts. Source data are provided as a Source Data file.

Supplementary Table 5. Fitted parameters for the lipid A: d-DPPC bilayer.

Layers	Thickness (Å)	d-DPPC (%)	Lipid A (%)	Hydration (%)
SiO_2	13.1 ± 0.1	-	-	-
d-DPPC head	12.1 ± 0.1	86.1 ± 0.8[a]		13.9 ± 7.7
d-DPPC tail	18.9 ± 0.1	88.2 ± 0.6	10.0 ± 0.1	1.8 ± 0.6
Lipid A tail	16.8 ± 0.1	12.3 ± 2.6	80.0 ± 1.6	7.7 ± 4.1
Lipid A head	8.1 ± 0.3	95.7 ± 0.8[a]		4.3 ± 1.9

[a]Total volume fraction of lipid A and d-DPPC head groups within the fitted layer, determined according to the minimal isotopic contrast between the head groups of lipid A and d-DPPC, while bilayer asymmetry was calculated according to the neutron SLD of the tail regions of lipid A and d-DPPC in three isotopic contrasts (D_2O, CMSi, and H_2O).

Supplementary Table 6. Fitted parameters for the lipid A: d-DPPC bilayer after exposure to 32 μg/ml cubosomes.

Layers	Thickness (Å)	d-DPPC (%)	Lipid A (%)	Phytantriol (%)	Hydration (%)
SiO_2	13.2 ± 0.1	-	-	-	-
d-DPPC head	12.1 ± 0.1	81.1 ± 0.3[a]		5.8 ± 1.3	13.1 ± 5.1
d-DPPC tail	18.5 ± 0.0	74.6 ± 1.1	20.4 ± 0.2[b]		5.0 ± 0.6
Lipid A tail	16.8 ± 0.1	12.5 ± 3.5	77.1 ± 0.4[b]		10.4 ± 1.8
Lipid A head	8.0 ± 0.3	80.9 ± 0.9[a]		6.5 ± 2.0	12.6 ± 6.0
Phytantriol	50.7 ± 0.1	-	-	17.5 ± 5.3	82.5 ± 5.3

[a]Total volume fraction of lipid A and d-DPPC head groups within the fitted layer, determined according to the minimal isotopic contrast between the head groups of lipid A and d-DPPC, while bilayer asymmetry was calculated according to the neutron SLD of the tail regions of lipid A and d-DPPC in three isotopic contrasts (D_2O, CMSi, and H_2O). [b]Total volume fraction of lipid A and cubosomes tail within the fitted layer, determined according to the minimal isotopic contrast between the tails of lipid A and phytantriol.

Supplementary Table 7. Fitted parameters for the cubosomes-treated lipid A: d-DPPC bilayer after exposure to 4 µg/ml PMB.

Layers	Thickness (Å)	d-DPPC (%)	Lipid A (%)	Phytantriol (%)	PMB (%)	Hydration (%)
SiO_2	13.1 ± 0.1	-	-	-	-	-
d-DPPC head	12.1 ± 0.1	80.6 ± 0.7[a]		7.5 ± 1.8	-	11.9 ± 3.5
d-DPPC tail	18.6 ± 0.1	74.0 ± 0.9	20.1 ± 0.1[b]		-	5.9 ± 0.7
Lipid A tail	16.9 ± 0.1	12.5 ± 3.5	76.9 ± 0.2[b]		-	10.6 ± 1.6
Lipid A head	8.0 ± 0.4	85.1 ± 1.1[a]		6.7 ± 0.9	-	8.2 ± 2.2
Phytantriol + PMB	37.8 ± 0.1	-	-	13.4 ± 3.5	14.0 ± 5.7	72.6 ± 4.1
PMB	12.3 ± 0.3	-	-	-	37.5 ± 7.0	62.5 ± 7.0

[a]Total volume fraction of lipid A and d-DPPC head groups within the fitted layer, determined according to the minimal isotopic contrast between the head groups of lipid A and d-DPPC, while bilayer asymmetry was calculated according to the neutron SLD of the tail regions of lipid A and d-DPPC in three isotopic contrasts (D_2O, CMSi, and H_2O). [b]Total volume fraction of lipid A and cubosomes tail within the fitted layer, determined according to the minimal isotopic contrast between the tails of lipid A and phytantriol.

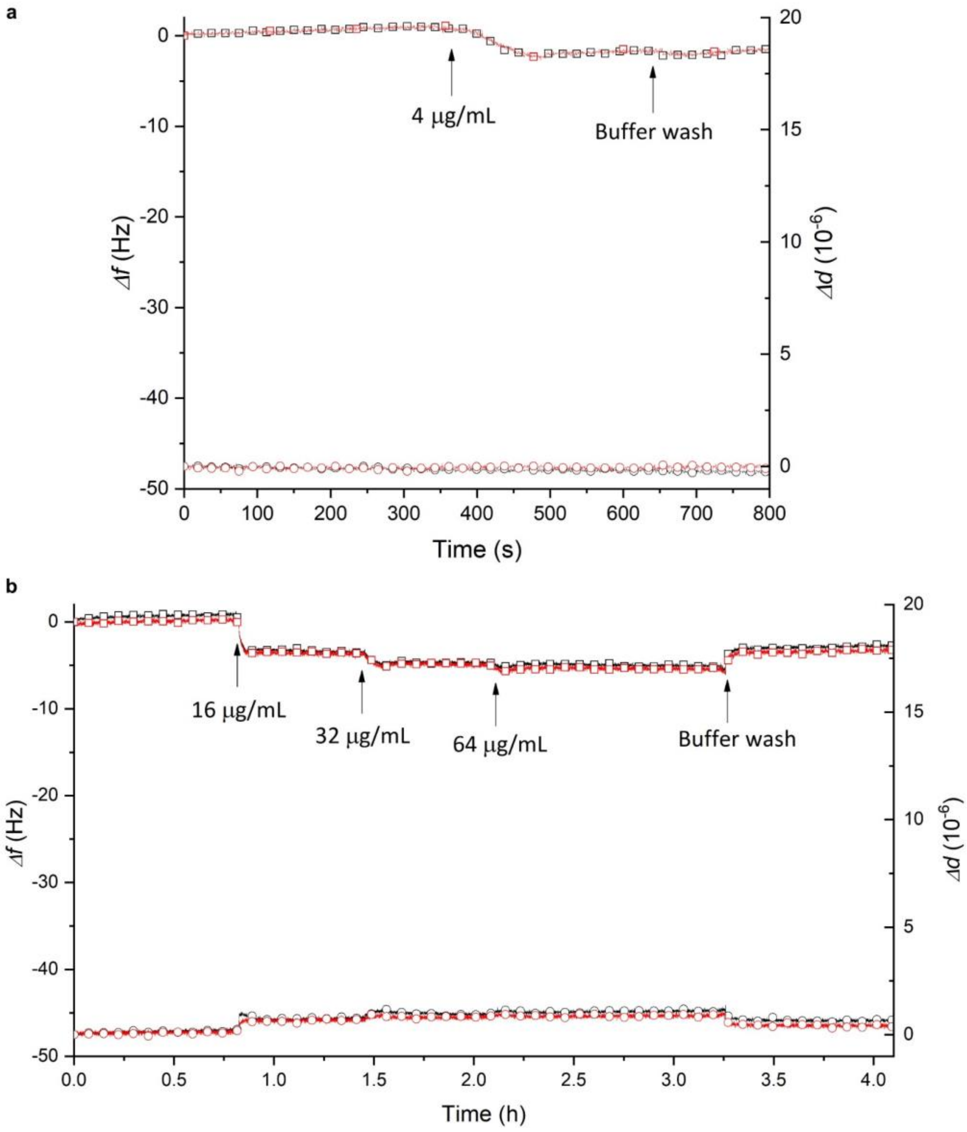

Supplementary Fig. 10. **Interaction between treatments and SiO2-coated QCM sensor surface.** Real-time QCM-D measurements for the interaction of (**a**) PMB and (**b**) cubosomes with SiO_2, respectively. Two overtones are shown for 3rd and 5th. Black squares: f_3; red squares: f_5; Black circles: d_3; Red circles: d_5; Buffer (5 mM CaCl2, 150 mM NaCl, and 10 mM HEPES, pH 7.4. The addition of 4 µg PMB (4 µg/mLx 200 µl /min injection rate x 5 min) resulted in a resonance frequency shift of only ~ 3 Hz, which indicated a relatively small mass change (~ 1.3 % PMB) on the SiO_2 surface. The addition of 16 µg/mL cubosomes for 5 min (flow rate at 200 µl/ml) leads to a frequency change of only ~ 4 Hz (~ 1.8 % PMB adsorption). Source data are provided as a Source Data file.

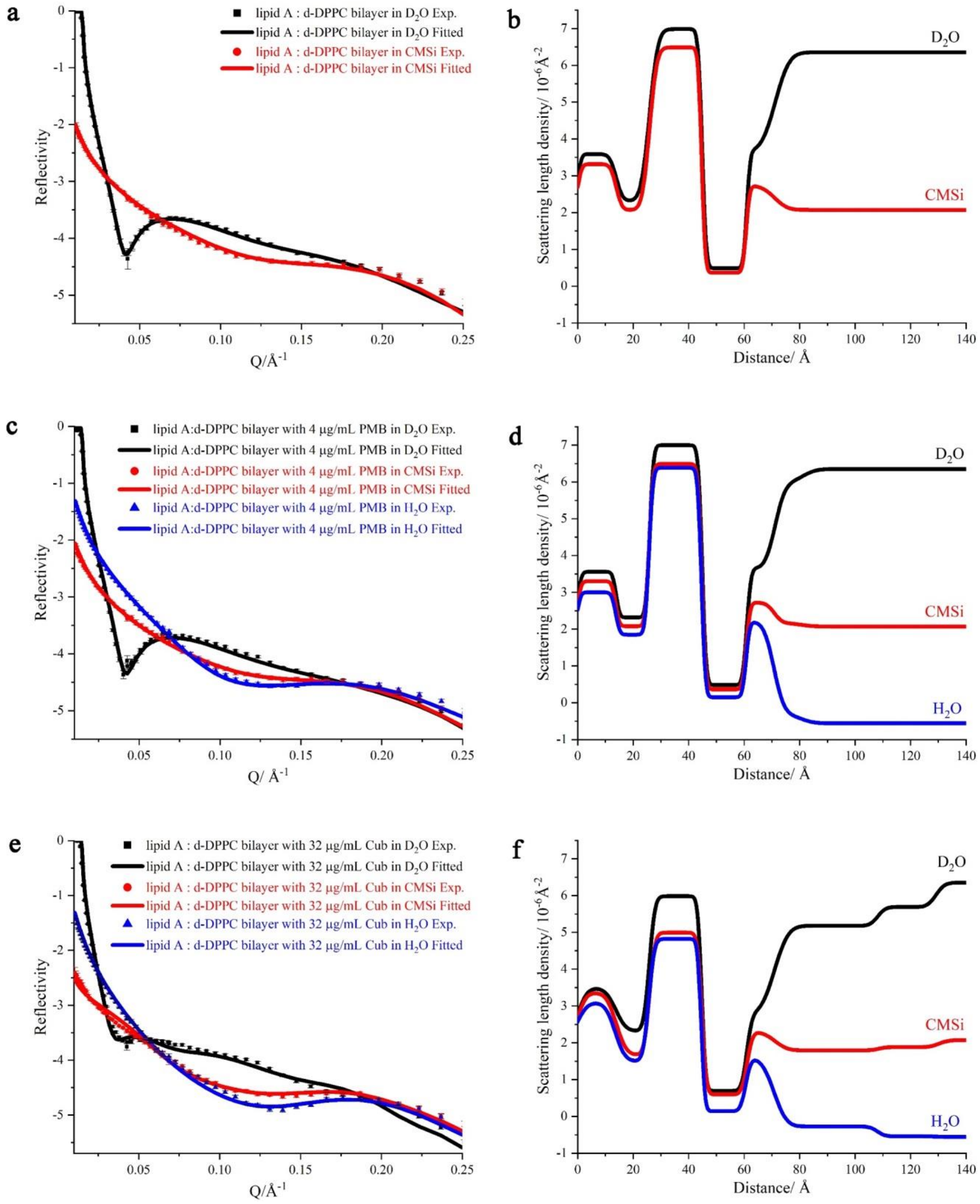

Supplementary Fig. 11. Lipid A: d-DPPC bilayer treated with 4 μg/mL PMB followed by 32 μg/mL cubosomes. Experimental (Exp., symbols) and fitted (solid lines) NR profiles in D_2O (black squares), CMSi (red circles) and H_2O (blue up triangle). **a**, lipid A: d-DPPC bilayer; **b**, the corresponding SLD profiles of (**a**); **c**, bilayer (**a**) treated with 4 μg/mL PMB; **d**, the corresponding SLD profiles of (**c**); **e**, followed by treatment with 32 μg/mL cubosomes (Cub); **f**, the corresponding SLD profiles of (**e**). For each condition in neutron reflectometry, we conducted multiple isotopic contrasts (D_2O, H_2O and CMSi) which would remove any artifacts in the fitting. The error bars are the standard deviation of neutron counts. Source data are provided as a Source Data file.

Supplementary Table 8. Fitted parameters for the lipid A: d-DPPC bilayer

Layers	Thickness (Å)	d-DPPC (%)	Lipid A (%)	Hydration (%)
SiO_2	13.9 ± 0.1	-	-	-
d-DPPC head	12.0 ± 0.1	96.3 ± 0.1[a]		3.7 ± 0.7
d-DPPC tail	19.4 ± 0.6	88.6 ± 1.6	8.2 ± 2.9	1.9 ± 0.1
Lipid A tail	16.1 ± 0.1	8.5 ± 0.5	88.7 ± 0.4	2.9 ± 0.9
Lipid A head	10.3 ± 0.1	89.4 ± 1.4[a]		10.6 ± 1.6

[a]Total volume fraction of lipid A and d-DPPC head groups within the fitted layer, determined according to the minimal isotopic contrast between the head groups of lipid A and d-DPPC, while bilayer asymmetry was calculated according to the neutron SLD of the tail regions of lipid A and d-DPPC in three isotopic contrasts (D_2O, CMSi, and H_2O).

Supplementary Table 9. Fitted parameters for the lipid A: d-DPPC bilayer after exposure to 4 µg/ml PMB.

Layers	Thickness (Å)	d-DPPC (%)	Lipid A (%)	PMB (%)	Hydration (%)
SiO_2	13.7 ± 0.1	-	-	-	-
d-DPPC head	12.1 ± 0.1	92.0 ± 1.9[a]		4.3 ± 1.2	5.6 ± 3.0
d-DPPC tail	18.9 ± 0.1	86.3 ± 1.3	5.7 ± 1.2	3.0 ± 1.4	5.0 ± 1.4
Lipid A tail	16.1 ± 0.1	6.0 ± 0.8	85.0 ± 1.6	4.8 ± 2.1	4.2 ± 2.7
Lipid A head	10.1 ± 0.2	86.1 ± 0.4[a]		2.7 ± 0.9	11.2 ± 1.4
PMB	9.8 ± 0.2	-	-	16.8 ± 3.2	83.2 ± 3.2

[a]Total volume fraction of lipid A and d-DPPC head groups within the fitted layer, determined according to the minimal isotopic contrast between the head groups of lipid A and d-DPPC, while bilayer asymmetry was calculated according to the neutron SLD of the tail regions of lipid A and d-DPPC in three isotopic contrasts (D_2O, CMSi, and H_2O).

Supplementary Table 10. Fitted parameters for the PMB-treated lipid A: d-DPPC bilayer after exposure to 32 µg/ml cubosomes

Layers	Thickness (Å)	d-DPPC (%)	Lipid A (%)	Phytantriol (%)	PMB (%)	Hydration (%)
SiO_2	13.7 ± 0.2	-	-	-	-	-
d-DPPC head	12.1 ± 0.1	66.3 ± 0.2[a]		20.3 ± 0.5	6.7 ± 0.9	6.7 ± 3.7
d-DPPC tail	18.9 ± 0.1	60.9 ± 1.4	16.7 ± 1.2[b]		9.0 ± 2.9	13.4 ± 6.5
Lipid A tail	16.2 ± 0.1	6.0 ± 0.8	83.0 ± 1.0[b]		6.8 ± 0.6	4.2 ± 2.5
Lipid A head	10.1 ± 0.1	64.9 ± 0.7[a]		20.2 ± 4.6	9.7 ± 0.5	5.2 ± 3.5
PMB + Phytantriol	37.2 ± 0.1	-	-	17.1 ± 2.4	14.0 ± 1.4	68.9 ± 1.3
Phytantriol	21.5 ± 0.3	-	-	12.5 ± 3.3	-	87.5 ± 3.3

[a]Total volume fraction of lipid A and d-DPPC head groups within the fitted layer, determined according to the minimal isotopic contrast between the head groups of lipid A and d-DPPC, while bilayer asymmetry was calculated according to the neutron SLD of the tail regions of lipid A and d-DPPC in three isotopic contrasts (D_2O, CMSi, and H_2O). [b]Total volume fraction of lipid A and cubosomes tail within the fitted layer, determined according to the minimal isotopic contrast between the tails of lipid A and phytantriol.

Supplementary Table 11. SLD used in the modelling of Bilayers

Materials	SLD (× 10^{-6} Å^{-2})		
	D_2O[a]	CMSi[b]	H_2O[c]
Si	2.07	2.07	2.07
SiO_2	3.41	3.41	3.41
H_2O	-	-	-0.56
CMSi		2.07	
D_2O	6.35	-	-
DPPC head	1.98	1.98	1.98
d-DPPC tail[d]	7.45	7.45	7.45
Lipid A tail	-0.39	-0.39	-0.39
Lipid A GlcN head	3.39	2.89	2.58
Polymyxin B	4.18	2.56	1.57
Phytantriol	0.365	-0.102	-0.388
F127	0.50	0.50	0.50

[a]D_2O 100%, offered maximum contrast with the hydrogenated lipid; [b]D_2O/H_2O (38/62, v/v), contrast matched the silicon substrate; [c]H_2O 100%, offered maximum contrast with the deuterated lipid. [d]The tails of DPPC were deuterated in order to offer enough isotopic contrast between the inner and outer leaflets under neutron measurements. GlcN, glucosamine.

Supplementary References

1 Gyulai, G. *et al.* Preparation and characterization of cationic Pluronic for surface modification and functionalization of polymeric drug delivery nanoparticles. *Express Polymer Letters* **10**, 216 (2016).

2 Yaghmur, A. *et al.* Oil-loaded monolinolein-based particles with confined inverse discontinuous cubic structure (Fd 3 m). *Langmuir* **22**, 517-521 (2006).

3 Silvestrini, A. V. P., Caron, A. L., Viegas, J., Praca, F. G. & Bentley, M. Advances in lyotropic liquid crystal systems for skin drug delivery. *Expert Opin Drug Deliv* **17**, 1781-1805 (2020).

4 Hyde, S. T. Bicontinuous structures in lyotropic liquid crystals and crystalline hyperbolic surfaces. *Curr. Opin. Solid State Mater. Sci.* **1**, 653-662 (1996).
