# Supplementary material for: A polytherapy based approach to combat antimicrobial resistance using cubosomes
Source: Nat Commun. 2022 Jan 17;13:343. doi: 10.1038/s41467-022-28012-5 (PMC8763928; doi:10.1038/s41467-022-28012-5)
Supplement: Supplementary file 3 — Description of Additional Supplementary Files [file 41467_2022_28012_MOESM3_ESM.docx]

**Title**: Supplementary Data 1.

**Description**: Minimum inhibitory concentration (MIC) of PMB, PMB-loaded cubosomes and FIC of PMB with cubosomes against isolates of *A. baumannii, P. aeruginosa, and K. pneumoniae* in DMEM/10 % FBS cell culture medium.
