# Supplementary material for: A polytherapy based approach to combat antimicrobial resistance using cubosomes
Source: Nat Commun. 2022 Jan 17;13:343. doi: 10.1038/s41467-022-28012-5 (PMC8763928; doi:10.1038/s41467-022-28012-5)
Supplement: Supplementary file 5 — Reporting Summary [file 41467_2022_28012_MOESM5_ESM.pdf]

nature portfolio

Corresponding author(s): Jian Li and Hsin-Hui Shen

Last updated by author(s): Nov 23, 2021

Reporting Summary

Nature Portfolio wishes to improve the reproducibility of the work that we publish. This form provides structure for consistency and transparency in reporting. For further information on Nature Portfolio policies, see our Editorial Policies and the Editorial Policy Checklist.

Statistics

For all statistical analyses, confirm that the following items are present in the figure legend, table legend, main text, or Methods section.

n/a	Confirmed	
☐	☒	The exact sample size (n) for each experimental group/condition, given as a discrete number and unit of measurement
☐	☒	A statement on whether measurements were taken from distinct samples or whether the same sample was measured repeatedly
☒	☐	The statistical test(s) used AND whether they are one- or two-sided<br>*Only common tests should be described solely by name; describe more complex techniques in the Methods section.*
☒	☐	A description of all covariates tested
☒	☐	A description of any assumptions or corrections, such as tests of normality and adjustment for multiple comparisons
☐	☒	A full description of the statistical parameters including central tendency (e.g. means) or other basic estimates (e.g. regression coefficient) AND variation (e.g. standard deviation) or associated estimates of uncertainty (e.g. confidence intervals)
☒	☐	For null hypothesis testing, the test statistic (e.g. F, t, r) with confidence intervals, effect sizes, degrees of freedom and P value noted<br>*Give P values as exact values whenever suitable.*
☒	☐	For Bayesian analysis, information on the choice of priors and Markov chain Monte Carlo settings
☒	☐	For hierarchical and complex designs, identification of the appropriate level for tests and full reporting of outcomes
☒	☐	Estimates of effect sizes (e.g. Cohen's d, Pearson's r), indicating how they were calculated

Our web collection on statistics for biologists contains articles on many of the points above.

Software and code

Policy information about availability of computer code

Data collection	Zetasizer Software V6.28, QSoft401 V2.5.2.418, Diffrac.Suite V 7.3.1, SparkControl V2.3 (Spark 10M), AnalySIS v3.2 camera control software (Olympus),
Data analysis	Originlab 2018 b9.5.1.195, Bruker Diffrac.SAXS 1.0, Microsoft 365 version 2105, GraphPad Prism version 9.0.1 (151), ImageJ 1.53a and Igor Pro 6.37 were used for data analysis

For manuscripts utilizing custom algorithms or software that are central to the research but not yet described in published literature, software must be made available to editors and reviewers. We strongly encourage code deposition in a community repository (e.g. GitHub). See the Nature Portfolio guidelines for submitting code & software for further information.

Data

Policy information about availability of data

All manuscripts must include a data availability statement. This statement should provide the following information, where applicable:

- Accession codes, unique identifiers, or web links for publicly available datasets
- A description of any restrictions on data availability
- For clinical datasets or third party data, please ensure that the statement adheres to our policy

The authors declare that the data supporting the findings of this study are available within the article and its Supplementary files. Source data are provided with this paper.

Field-specific reporting

Please select the one below that is the best fit for your research. If you are not sure, read the appropriate sections before making your selection.

☒ Life sciences ☐ Behavioural & social sciences ☐ Ecological, evolutionary & environmental sciences

For a reference copy of the document with all sections, see nature.com/documents/nr-reporting-summary-flat.pdf

Life sciences study design

All studies must disclose on these points even when the disclosure is negative.

Sample size	No statistical method was used to determine the sample size, and n=3 was used because we can see the significant effects
Data exclusions	No data were excluded.
Replication	All experiments except neutron reflectometry were independently repeated three times. All attempts to replicate the experiments were successful. For each condition in neutron reflectometry, we conducted multiple isotopic contrasts (D2O, H2O, CMSi) which would remove any artifacts in the fitting as it is unlikely that a wrong model would be able to fit all contrasts adequately.
Randomization	All the bacteria strains were randomly selected from their corresponding culture collection.
Blinding	All strains were randomly selected without analyzing the genetic and genomic information of the strains.

Reporting for specific materials, systems and methods

We require information from authors about some types of materials, experimental systems and methods used in many studies. Here, indicate whether each material, system or method listed is relevant to your study. If you are not sure if a list item applies to your research, read the appropriate section before selecting a response.

Materials & experimental systems

n/a	Involved in the study	
☒	☐	Antibodies
☐	☒	Eukaryotic cell lines
☒	☐	Palaeontology and archaeology
☒	☐	Animals and other organisms
☒	☐	Human research participants
☒	☐	Clinical data
☒	☐	Dual use research of concern

Methods

n/a	Involved in the study	
☒	☐	ChIP-seq
☒	☐	Flow cytometry
☒	☐	MRI-based neuroimaging

Eukaryotic cell lines

Policy information about cell lines

Cell line source(s)	ATCC HEK-293T cell line was the gift from Thomas Naderer (Department of Biochemistry and Molecular Biology, Monash University, Australia)
Authentication	None of the cell lines were authenticated.
Mycoplasma contamination	Cells were negative from mycoplasma contamination
Commonly misidentified lines (See ICLAC register)	No cell lines used in this study were found in the database of commonly misidentified cell lines that is maintained by ICLAC register.
